# Estrogen receptor-α-miR-1271-SNAI2 feedback loop regulates transforming growth factor-β-induced breast cancer progression

**DOI:** 10.1186/s13046-019-1112-4

**Published:** 2019-03-01

**Authors:** Bo-Wen Liu, Zhi-Hao Yu, Ao-Xiang Chen, Jiang-Rui Chi, Jie Ge, Yue Yu, Xu-Chen Cao

**Affiliations:** 1 The First Department of Breast Cancer, National Clinical Research Center for Cancer, Tianjin Medical University Cancer Institute and Hospital, Huan-Hu-Xi Road, Hexi District, Tianjin, 300060 China; 2 Key Laboratory of Cancer Prevention and Therapy, Tianjin, 300060 China; 3 Tianjin’s Clinical Research Center for Cancer, Tianjin, 300060 China; 4 Key Laboratory of Breast Cancer Prevention and Therapy, Tianjin Medical University, Ministry of Education, Tianjin, 300060 China

**Keywords:** Breast cancer, Transforming growth factor-β, Epithelial to mesenchymal transition, miR-1271, SNAI2, ERα

## Abstract

**Background:**

Breast cancer is the most common cancer among women worldwide, and approximately 70% of breast cancers are hormone receptor-positive and express estrogen receptor-α (ERα) or/and progesterone receptor. ERα has been identified to promote the growth of primary breast cancer, however, it can also antagonize signaling pathways that lead to epithelial-mesenchymal transition (EMT), including transforming growth factor-β (TGF-β) signaling. miRNA alteration or dysfunction is involved in cancer development and progression. Although miR-1271 has identified as a tumor suppressor in various cancers, the role of miR-1271 in breast cancer is still limited.

**Methods:**

The effect of miR-1271 on breast cancer progression was investigated both in vitro and in vivo. The EMT-related protein expression levels and localization were analyzed by western blotting and immunofluorescence, respectively. Chromatin immunoprecipitation and dual-luciferase reporter assays were used to validate the regulation of ERα-miR-1271-SNAI2 feedback loop.

**Results:**

miR-1271 suppresses breast cancer progression and EMT phenotype both in vitro and in vivo by targeting SNAI2. Estrogen reverses TGF-β-induced EMT in a miR-1271 dependent manner. Furthermore, ERα transactivates the miR-1271 expression and is also transcriptionally repressed by SNAI2.

**Conclusions:**

Our data uncover the ERα-miR-1271-SNAI2 feedback loop and provide a mechanism to explain the TGF-β network in breast cancer progression.

**Electronic supplementary material:**

The online version of this article (10.1186/s13046-019-1112-4) contains supplementary material, which is available to authorized users.

## Introduction

Breast cancer is the most frequently diagnosed malignancy in women worldwide [1]. It is the most common malignant tumor, and the third largest cause of cancer-related deaths in China. Although the incidence of this disease is increasing, the number of deaths caused by it is decreasing [2]. Different therapeutic strategies are applied according to the molecular subtypes and patients have greatly benefited from molecular classification.

Approximately 70% of breast cancers are hormone receptor-positive and express estrogen receptor-α (ERα) or/and progesterone receptor. ERα can both activate and repress the expression of down-stream target genes as a ligand-activated transcription factor and is a key regulator of breast cancer development and progression. In addition, ERα serves as an important prognostic factor in patients with breast cancer [3]. ERα has been identified to promote the growth of primary breast cancer, however, it can also antagonize signaling pathways that lead to epithelial-mesenchymal transition (EMT), including transforming growth factor-β (TGF-β) signaling [4, 5]. The TGF-β signaling pathway induces breast cancer progression by regulation of multiple stages in the metastatic process, among which EMT is a well-studied process that endows tumor cells with increased aggressiveness [6]. Thus, ERα depletion is closely related to more serious malignant phenotypes and an improved understanding of the ERα-TGF-β regulation network may reveal new strategies for breast cancer therapy.

miRNAs are a class of small, endogenous, non-coding RNAs that negatively regulate the expression of a wide variety of genes by binding to complementary sequences in the 3′-untranslated regions (UTRs) of target mRNAs [7, 8]. A large number of studies have shown that miRNA alteration or dysfunction is involved in cancer development and progression by regulating cancer cell proliferation, differentiation, apoptosis, angiogenesis, metastasis, and metabolism [9, 10]. Dysregulated miRNAs are involved in breast cancer carcinogenesis and progression and function as oncogenes or tumor suppressors, as well as useful biomarkers in the diagnosis and prognosis of breast cancer [11, 12]. However, our understanding of how miRNAs regulate breast cancer development and progression is still limited. miR-1271 is located at an intron region of the ADP ribosylation factor like GTPase 10 gene on chromosome 5q35.2. To date, many studies have demonstrated that decreased expression of miR-1271 is associated with the development and progression of various human cancers, including hepatocellular carcinoma [13–15], gastric cancer [16, 17], colorectal cancer [18, 19], pancreatic cancer [20, 21], lung cancer [22, 23], glioma [24, 25], osteosarcoma [26], prostate cancer [27], endometrial cancer [28], myeloma [29], oral squamous cell carcinoma [30], ovarian cancer [31] and breast cancer [32, 33]. Recently, although one study indicated that miR-1271 functions as a tumor suppressor via targeting SPIN1, the mechanism of miR-1271 in breast cancer development and progression is still limited [32].

Here, we investigate the role of miR-1271 in breast cancer development and progression. ERα antagonizes TGF-β-induced EMT by transactivation of miR-1271 expression in an estrogen-dependent manner. We further demonstrated that the zinc-finger transcription factor Snail family transcriptional repressor 2 (SNAI2) is a target of miR-1271 and transcriptionally suppresses the expression of ERα. Therefore, our study reveals an ERα-miR-1271-SNAI2 feedback loop in regulation of TGF-β signaling during breast cancer development and progression.

## Materials and methods

### Cell culture and human specimens

The human breast cancer cell lines MCF7, T47D, BT474, BT549, MDA-MB-468, and MDA-MB-231 cell lines were obtained from the Cell Bank of the Chinese Academy of Sciences (Shanghai, China) and cultured as previously described [6].

Breast cancer specimens were obtained from Tianjin Medical University Cancer Institute and Hospital (TMUCIH). Thirty primary breast cancer tissue samples were used for this study. All tumor samples were obtained from patients newly diagnosed with breast cancer and who had received no therapy before sample collection. This study was approved by the Institutional Review Board of TMUCIH and written consent was obtained from all participants.

### Antibodies and reagents

Antibodies against N-cadherin, E-cadherin, vimentin, HA (Santa Cruz Biotechnology, Santa Cruz, CA, USA), SNAI2 (Abcam, Cambridge, MA, USA), pSMAD2, ERα and β-actin (Cell Signaling Technology, Beverly, MA, USA) were used. Recombinant human TGF-β1 was purchased from R&D Systems (Redmond, WA, USA). Insulin and E2 were purchased from Sigma-Aldrich (St Louis, MO, USA).

### Plasmids, miRNA, small interfering RNA (siRNA) and transfection

The ORF of human SNAI2 and ERα generated from MDA-MB-231 and MCF7 cells, the resultant PCR product of which was connected together with pcDNA3.1 tagged HA. The SNAI2 3′-UTR containing miR-1271 binding sites were amplified and cloned into psiCHECK2 vector (Promega, Madison, WI, USA) to generate Luc-SNAI2. Site-directed mutagenesis was performed using the Site-Directed Mutagenesis Kit (TransGene, Beijing, China) to generate the SNAI2 3′-UTRmut reporter vector (SNAI2M). The miR-1271 promoter region (P1: − 1100 to + 1; P2: -705 to + 1), Estrogen Receptor 1 (ESR1) promoter region (− 1000 to + 1) or the E-box mutated fragments were cloned into pGL3-Basic vector (Promega; pGL3-P1/P2 and pGL3-ESR1-wt/mut). All constructs were confirmed by sequencing. The miR-1271 mimic, miR-1271 inhibitor, or the appropriate scrambled controls were purchased from RiboBio (Shanghai, China). The ERα and SNAI2 gene-specific siRNAs, and non-specific control siRNA were also purchased from RiboBio.

miRNA or siRNAs were transfected into different cell lines using FuGENE HD Transfection Reagent (Promega, Madison, WI, USA) and plasmids were transfected using TransFast Transfection Reagent (Promega) according to the manufacturer’s recommendations.

### Migration and invasion assays

The invasive abilities of breast cancer cells in vitro were evaluated by Matrigel-coated Transwell (BD Biosciences, San Diego, CA, USA). Briefly, 5 × 10^4^ cells in 500 μL serum-free medium were added to the upper chamber, and medium containing 20% FBS was added into the lower chamber. Twenty-four hours later, the migrant cells that had attached to the lower surface were fixed with 20% methanol and stained for 20 min with crystal violet. The membranes were then carved and embedded under coverslips with the cells on the top. The number of migrating cells was counted under a microscope in five predetermined fields.

### Western blotting and immunofluorescence

Cells were lysed in protein lysis buffer [20 mM Tris-HCl (pH 7.4), 5 mM EDTA, 1% Triton X-100, 150 mM NaCl, and 1% DTT] containing a protease inhibitor cocktail tablet (Roche Molecular Biochemicals, Indianapolis, IN, USA). Proteins were resolved by sodium dodecyl sulfate-polyacrylamide gel electrophoresis, transferred onto polyvinylidene fluoride membranes (Millipore, Bedford, MA, USA), and incubated with primary antibodies overnight at 4 °C, followed by incubation with horseradish peroxidase-conjugated secondary antibody. The blots were visualized with ECL reagent (Millipore).

For immunofluorescence analysis, cells were seeded onto glass coverslips in 24-well plates, washed with phosphate buffered saline (PBS), fixed in 4% formaldehyde solution for 30 min, and then permeabilized with 0.2% Triton X-100/PBS for 15 min. Cells were blocked with 2% bovine serum albumin in PBS for 30 min. Coverslips were incubated with primary antibodies overnight at 4 °C, followed by incubation with FITC−/TRITC-conjugated secondary antibodies for 1 h at room temperature and then stained with 4′,6-diamidino-2-phenylindole. Finally, coverslips were observed under a fluorescence microscope.

### RNA extraction and reverse transcription quantitative polymerase chain reaction (RT-qPCR)

Total RNA of cultured cells, surgically resected fresh breast tissues, and formalin-fixed paraffin-embedded clinical specimens were extracted using mirVana PARIS kit (Life Technologies) according to the manufacturer’s recommendations. qPCR was performed to detect mRNA expression using GoTaq qPCR Master Mix (Promega). TaqMan RT-qPCR was performed to detect mature miRNA expression using TaqMan miRNA reverse transcription kit, hsa-RNU6B (U6, ABI Assay ID: 001093) and miR-1271 (ABI Assay ID: 002779) according to the manufacturer’s protocol (Life Technologies). The sequences of PCR primers were as previously described [6].

### Chromatin immunoprecipitation (ChIP) analysis

ChIP assay was performed according to the protocol of Upstate Biotechnology as previously described [34]. The primer sequences used for miR-1271 or ESR1 promoter were: 5′-CCAAGCACTTGACGGGCAT-3′ and 5′-CGACAGAGACCCTGTCTCC-3′ (miR-1271) or 5′-GGGCTAACTGATAAGTATAG-3′ and 5′-ATCTCATCATGTCTGGTGTG-3′ (ESR1). Monoclonal antibody against ERα or SNAI2 was used for immunoprecipitation and normal IgG was used as negative control. Five percent of original DNA was used as input control.

### Luciferase reporter assays

Luciferase assays were carried out using a dual luciferase assay kit according to the manufacturer’s recommendations as previously described [35].

### Xenograft

Stable miR-1271-overexpressing MDA-MB-231 and control cells (1 × 10^6^ cells) together with 100 μg of Matrigel (BD Biosciences, San Diego, CA, USA) were inoculated into the mammary fat pads of 5-week-old female BALB/c mice. Tumor growth was recorded once a week with a caliper-like instrument. Tumor volume was calculated according to the formula volume = (width^2^ × length)/2. Six weeks after inoculation, mice were killed, and the final volume and weight of tumor tissues were determined. All animal experimental protocols were approved by the Animal Ethics Committee of TMUCIH.

### Statistical analysis

Data are presented as mean ± standard deviation. The student’s *t*-test (2-tailed) was used to determine differences between the experimental and control groups. The level of significance was set to *P* < 0.05. All calculations were performed with the SPSS for Windows statistical software package (SPSS Inc., Chicago, IL, USA).

## Results

### miR-1271 predicts favorable outcome in patients with luminal a breast cancer

**Fig. 1:**
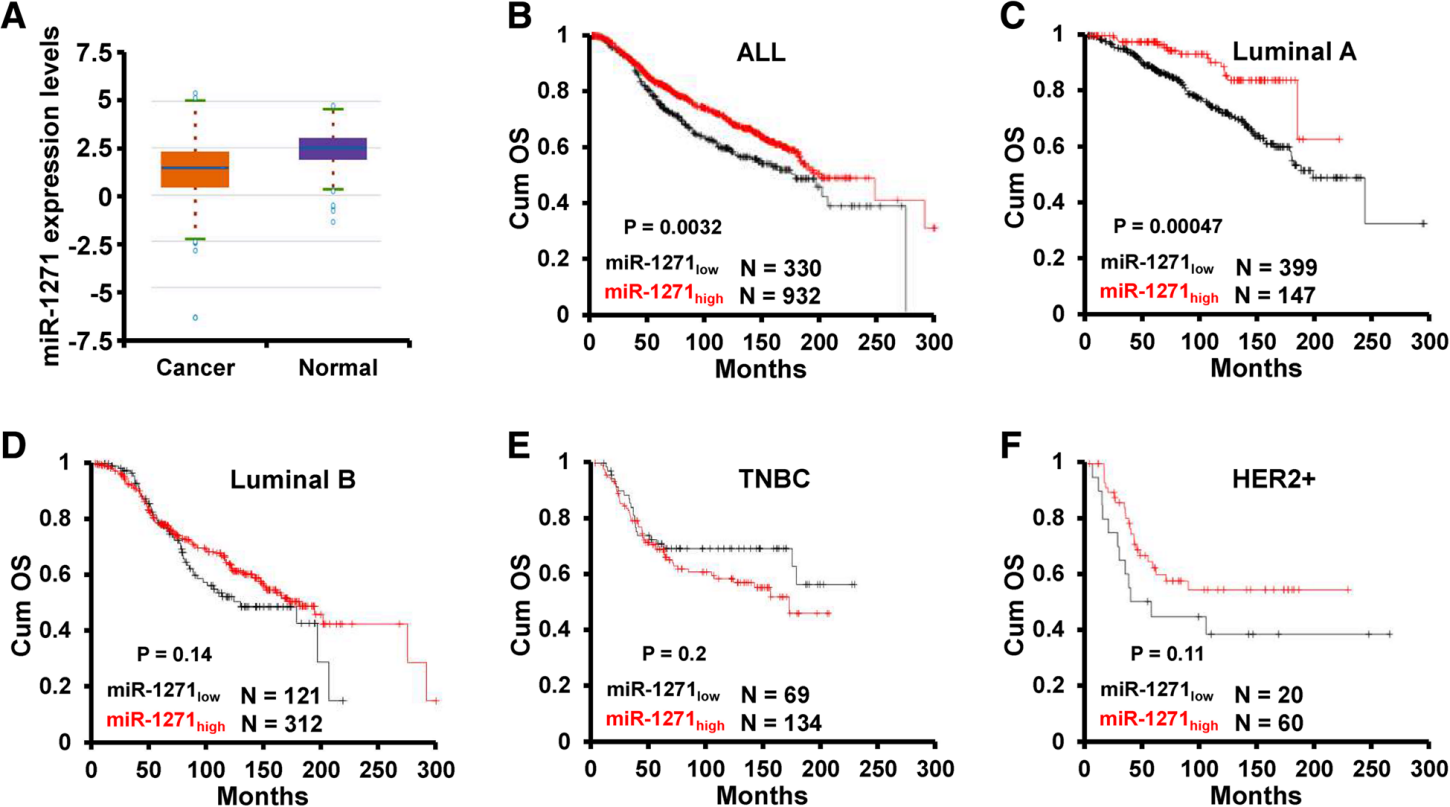
miR-1271 predicts favorable outcome in patients with Luminal A breast cancer. **a** The expression of miR-1271 in TCGA breast invasive carcinoma and normal tissues. **b-f,** Kaplan-Meier analysis of the OS in patients with different miR-1271 expression levels as determined using KM plotter. All patients (**b**), Luminal A (**c**), Luminal (**d**), TNBC (**e**), HER2+ (**f**)

We first examined the clinical relevance of miR-1271 expression in breast cancer progression. We analyzed the expression of miR-1271 in clinical breast cancer samples of The Cancer Genome Atlas (TCGA) database and found that miR-1271 expression was significantly lower in breast cancer tissues than in normal tissues (Fig. 1a). Furthermore, we compared the overall survival (OS) of breast cancer patients with different levels of miR-1271 expression by KM plotter (http://kmplot.com/analysis) and found that patients with high miR-1271 expression had a significantly favorable OS compared to those with low miR-1271 expression (Fig. 1b). More importantly, we found that miR-1271_high_ Luminal A breast cancer patients have favorable outcome compared to miR-1271_low_ patients (Fig. 1c). However, the expression of miR-1271 was not associated with the outcome in patients with Luminal B (Fig. 1d), TNBC (Fig. 1e), and HER2+ (Fig. 1f) breast cancer. Together, these results suggest that miR-1271 as a predictor in patients with Luminal A breast cancer.

### Down-regulation of miR-1271 is required for cell invasion through regulating EMT

**Fig. 2:**
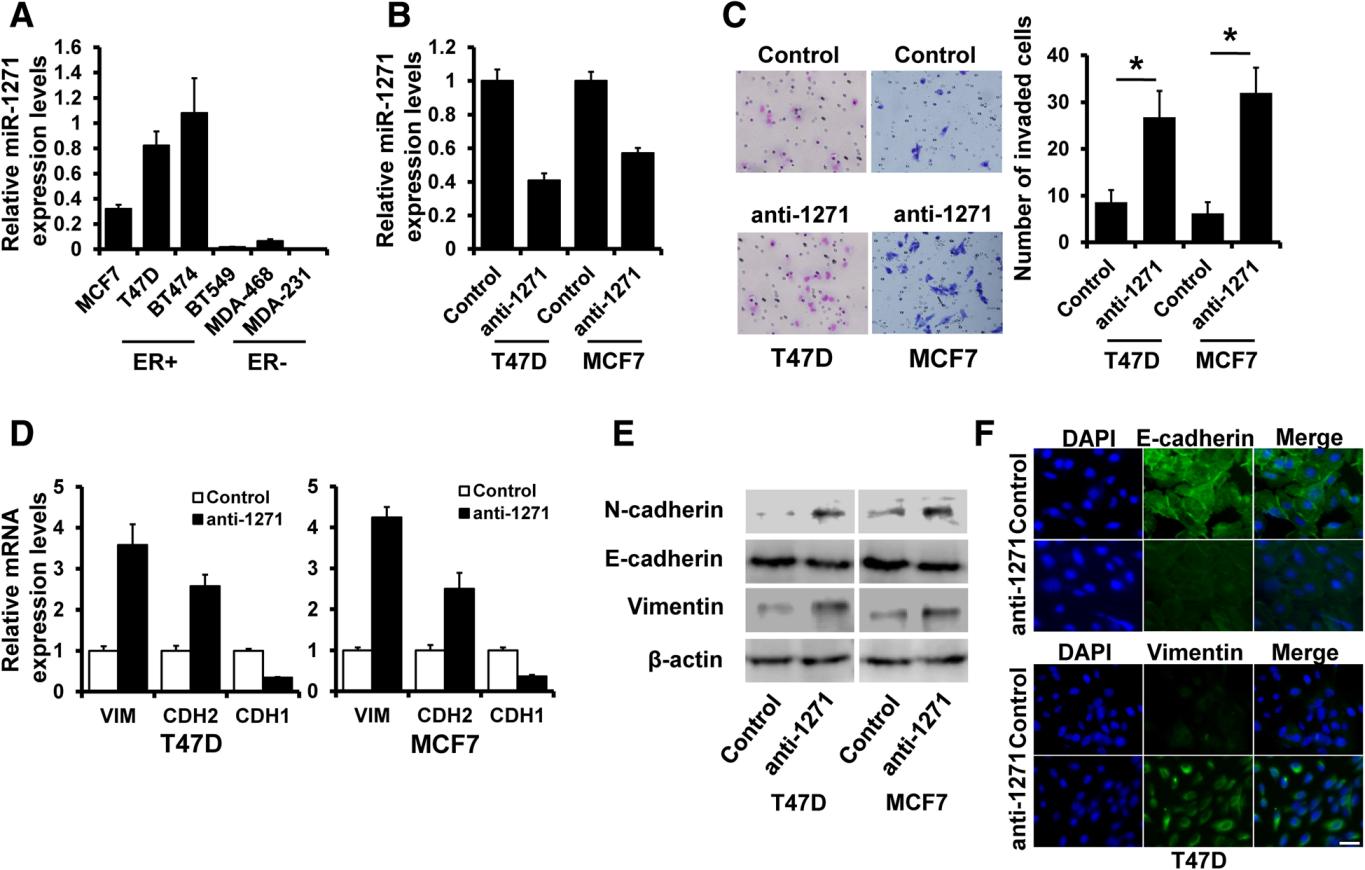
Depletion of miR-1271 inhibits cells invasion and EMT phenotype in Luminal A breast cancer. **a** The expression of miR-1271 in breast cancer cell lines as determined by RT-qPCR. **b** The expression of miR-1271in T47D and MCF7 cells transfected with miR-1271 inhibitor as determined by RT-qPCR. **c** Transwell invasion of cells as in (B). **d** and **e,** The mRNA (**d**) and protein (**e**) expression of EMT markers in cells as in (**b**) were detected by RT-qPCR and western blotting. **f** Immunofluorescence analyses of EMT markers in T47D cells. *P < 0.05. Scale bar, 50 μM

To investigate the role of miR-1271 in breast cancer progression, we determined the expression of miR-1271 in breast cancer cell lines (MCF7, T47D, BT474, BT549, MDA-MB-468, and MDA-MB-231) by RT-qPCR. We observed a high miR-1271 expression level in ER-positive breast cancer cell lines and a low expression level in ER-negative breast cancer cell lines (Fig. 2a). Next, we investigated the influence of miR-1271 on cell invasion and proliferation by transfecting T47D and MCF7 cells with miR-1271 inhibitor (Fig. 2b). Depletion of miR-1271 did not affect cell proliferation (Additional file 1: Figures S1A and B), but promoted cell invasion in both T47D and MCF7 cells by transwell assay (Fig. 2c). EMT plays a pivotal role during malignant tumor progression and metastasis, and miR-1271 functions as an EMT inhibitor in several types of cancers [13, 17, 21]. Thus, we next determined whether or not miR-1271 affects breast cancer progression through regulation of EMT. We examined both epithelial and mesenchymal marker expression by RT-qPCR (Fig. 2d), western blotting (Fig. 2e), and immunofluorescence (Fig. 2f). As can be seen, the miR-1271-depleted T47D or MCF7 cells showed significant down-regulation of E-cadherin, while the mesenchymal markers, vimentin and N-cadherin, were dramatically up-regulated (Fig. 2d, e, and f). Together, these results suggest that miR-1271 suppresses EMT and breast cancer progression.

### Overexpression of miR-1271 suppresses TNBC metastasis both in vitro *and* in vivo

**Fig. 3:**
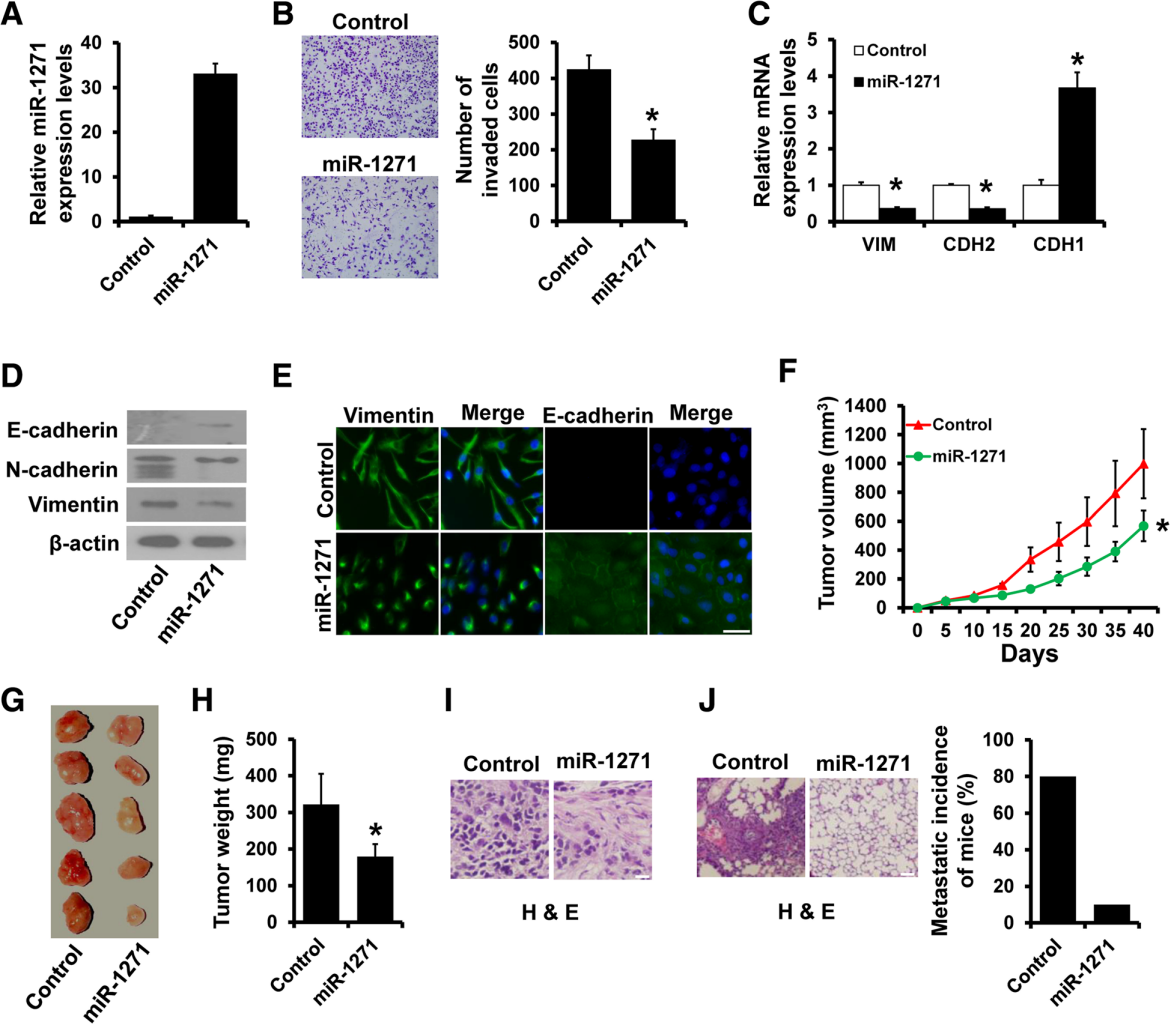
miR-1271 suppresses tumor growth and metastasis in TNBC. **a** The expression of miR-1271 in MDA-MB-231 cells with stable overexpression of miR-1271 as determined by RT-qPCR. **b** Transwell invasion assay of cells as in (A). **c** and **d,** The mRNA (C) and protein (D) expression of EMT markers in cells as in (A) were detected by RT-qPCR and western blotting. **e** Immunofluorescence analyses of EMT markers in cells as in (**a**). **f** Tumor volume of xenograft mice injected with MDA-MB-231-miR-1271 or control cells at the indicated times. **g** Representative photos of the tumors formed by MDA-MB-231 -miR-1271 or control cells at harvest time. **h** The weights of tumors formed by MDA-MB-231-miR-1271 or control cells at harvest time. **i** H&E staining in primary tumors harvested from mice bearing the indicated xenograft tumors. **j** Representative H&E lung images show lower number of lung nodules in lungs of mice injected with MDA-MB-231-miR-1271 cells compared to control. *P < 0.05. Scale bar, 50 μM

To investigate the role of miR-1271 in TNBC metastasis, we generated stable miR-1271-expressing cells by lentiviral infection of MDA-MB-231 cells (Fig. 3a). The MTT and colony formation assays also indicated that overexpression of miR-1271 did not affect MDA-MB-231 cell proliferation in vitro (Additional file 1: Figures S1C and 1D). The ability of cell invasion was significantly decreased in miR-1271-expressing MDA-MB-231 cells (Fig. 3b). Furthermore, we observed an increased E-cadherin expression and decreased vimentin and N-cadherin expression in miR-1271-expressing MDA-MB-231 cells (Fig. 3c-3e). Next, MDA-MB-231 cells stably expressing miR-1271 and control-transfected cells were implanted into the mammary fat pads of need mice, and tumor growth and metastasis were quantified. Overexpression of miR-1271 in MDA-MB-231 cells significantly inhibits tumor growth in vivo (Fig. 3f-h). In addition, H&E staining of the xenograft tissues showed less cell mitosis in tumors from the miR-1271expressing MDA-MB-231 cells than in tumors from the control cells (Fig. 3i). Furthermore, visible lung metastatic nodules were observed in 80% of the 231-control mice (4/5), whereas only one was observed in the lung of 231-miR-1271 mice (Fig. 3j). Together, these results indicate that overexpression of miR-1271 suppresses TNBC metastasis both in vitro and in vivo.

### Estrogen reverses TGF-β-induced EMT in a miR-1271 dependent manner

**Fig. 4:**
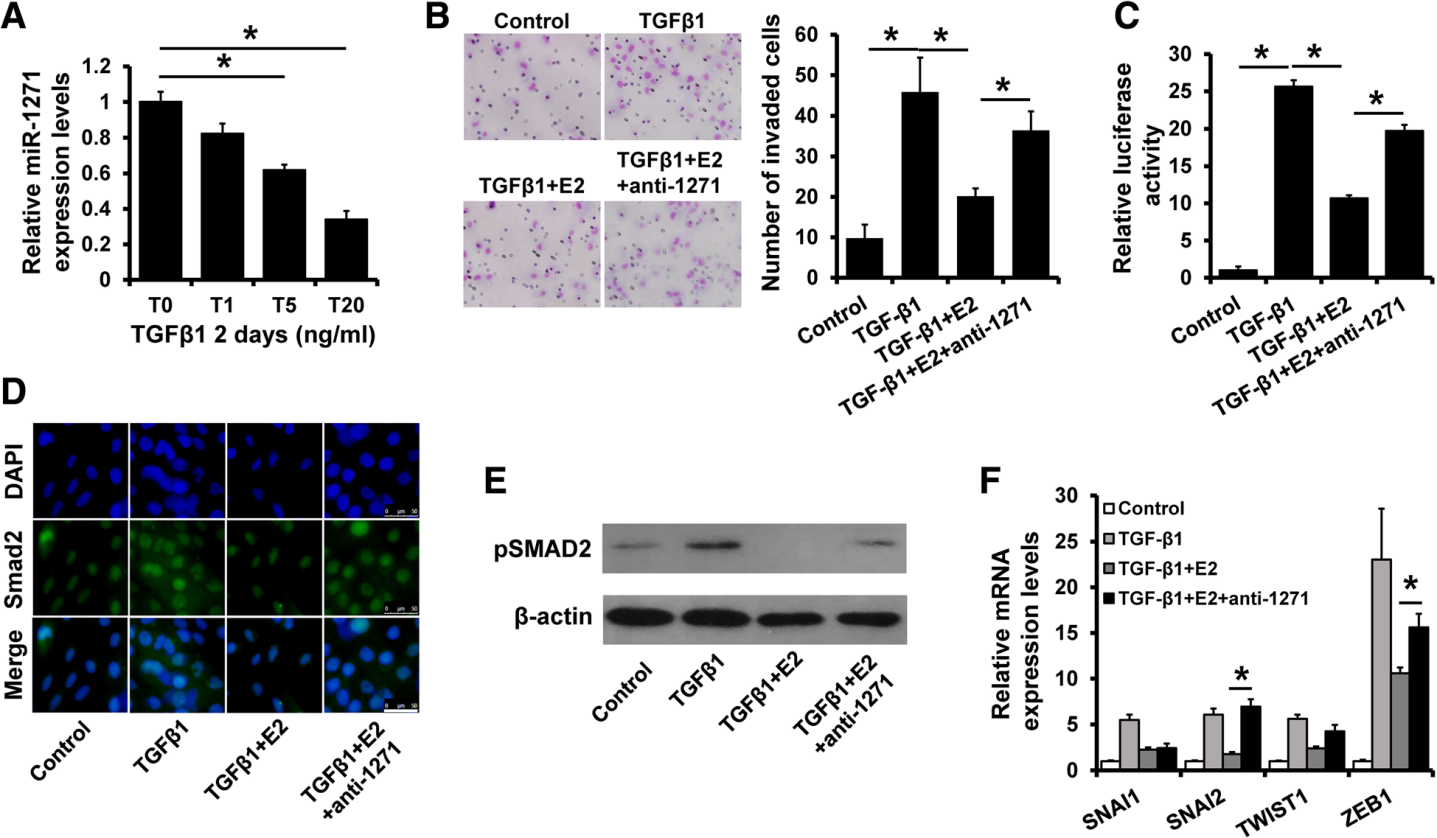
Estrogen reverses TGF-β-induced EMT in a miR-1271 dependent manner. **a** The expression of miR-1271 in T47D cells after treatment with TGF-β1 at indicated concentration. **b** Transwell invasion assay of miR-1271-depleted T47D or control cells with TGF-β1 or/and E2 treatment. **c** Luciferase reporter analysis of TGF-β signaling activity in cells as in (B). **d** Localization of SMAD2 in cells as in (B) as determined by immunofluorescence staining. **e** The expression of pSMAD2 in cells as in (A) by western blotting. **f** The expression of SNAI1/2, TWIST1 and ZEB1 in cells as in (A) as determined by RT-qPCR. *P < 0.05. Scale bar, 50 μM

TGF-β is a major inducer of EMT in development, carcinogenesis, and fibrosis. Previous study indicated that ERα suppresses breast cancer progression by inhibition of TGF-β signaling in an estrogen-dependent manner [4, 36]. Thus, we speculated that miR-1271 is involved in the suppressive effect of ERα and estrogen on TGF-β-induced breast cancer progression. We observed that miR-1271 expression was decreased in T47D cells after addition of TGF-β1 to the cell culture medium for 2 days (Fig. 4a). As shown in Fig. 4b, the invasive ability of T47D cells was dramatically increased after treatment with TGF-β1. In contrast, E2 treatment decreased the invasive ability of TGF-β1-treated T47D cells, whereas depletion of miR-1271 eliminates the effect of E2 on cell invasion (Fig. 4b). To further explore the role of miR-1271 in TGF-β signaling, we detected the luciferase activity of SMAD reporter in miR-1271-depleted T47D and control cells with or without TGF-β1 or E2 treatment. The luciferase activity was dramatically decreased in TGF-β1-treated cells after treatment with E2, but this effect was reversed in miR-1271-depleted T47D cells (Fig. 4c). The nuclear localization of SMAD2 and the expression of pSMAD2 was also increased in TGF-β1 and E2-treated T47D cells after transfection with miR-1271 inhibitors (Fig. 4d and e). We next investigated whether miR-1271 is involved in the suppressive effect of E2 on TGF-β signaling by regulating the expression of EMT-TFs, including SNAI1, SNAI2, TWIST1 and ZEB1. The expression of SNAI2 and ZEB1 was increased in TGF-β1 and E2-treated T47D cells after transfection with miR-1271 inhibitors by RT-qPCR (Fig. 4f). These results suggest that miR-1271 reverses the suppressive effect of estrogen on TGF-β-induced EMT.

### miR-1271 inhibits EMT by targeting SNAI2

**Fig. 5:**
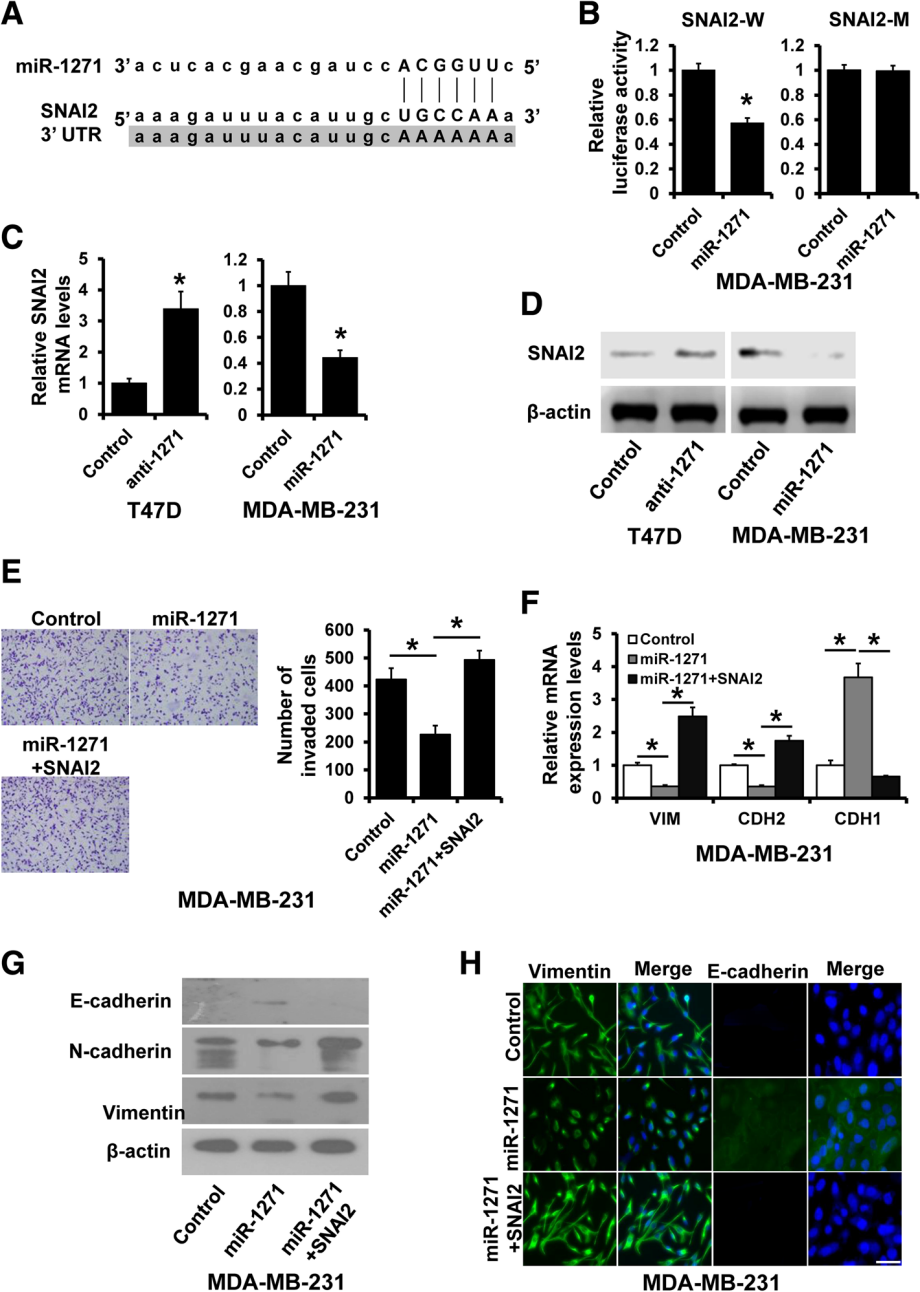
miR-1271 inhibits cell invasion and EMT by targeting SNAI2. **a** The predicted binding of miR-1271 with SNAI2 3′-UTR. **b** Dual luciferase reporter assay was performed to validate the miR-1271 target, SNAI2. **c** The expression of miR-1271 in indicated cells as determined by RT-qPCR. **d** The expression of SNAI2 in indicated cells as determined by western blotting. **e** Transwell invasion assay of miR-1271-overexpression MDA-MB-231 transfected with SNAI2 plasmid, as well as in control cells. **f** and **g** The mRNA (F) and protein (G) expression of EMT markers in cells as in (**e**) were detected by RT-qPCR and western blotting. **h** Immunofluorescence analyses of EMT markers in cells as in (**e**). *P < 0.05. Scale bar, 50 μM

The target prediction program, TargetScan, was applied to identify SNAI2 as a putative miR-1271 target (Fig. 5a). To further confirm this regulation, SNAI2 3′-UTR and its mutant containing the putative miR-1271 binding sties were cloned into the downstream luciferase ORF. As compared to control cells, the luciferase activity was significantly decreased in miR-1271-expressing MDA-MB-231 cells with inhibition rates of 50% (Fig. 5a left). These effects were abolished in mutated SNAI2 3′-UTR, in which the binding sites for miR-1271 were inactivated (SNAI2-M) by site-directed mutagenesis (Fig. 5b right). The expression of SNAI2 was increased in miR-1271-depleted T47D cells and was decreased in miR-1271-expressing MDA-MB-231 cells compared with that of control cells, as determined by RT-qPCR (Fig. 5c) and western blotting (Fig. 5d). To corroborate that SNAI2 mediates the role of miR-1271 in breast cancer progression, we transfected HA-SNAI2 plasmid into miR-1271-overexpressed MDA-MB-231 cells. SNAI2 overexpression abolished the effects of miR-1271 on MDA-MB-231 cell invasion (Fig. 5e). Transient transfection of HA-SNAI2 restored the EMT phenotypes in miR-1271-expressing MDA-MB-231 cells by RT-PCR (Fig. 5f), western blotting (Fig. 5g) and immunofluorescence staining (Fig. 5h). Thus, these results indicated that miR-1271 inhibits EMT and breast cancer invasion by suppressing SNAI2.

### ERα transactivates miR-1271 expression

**Fig. 6:**
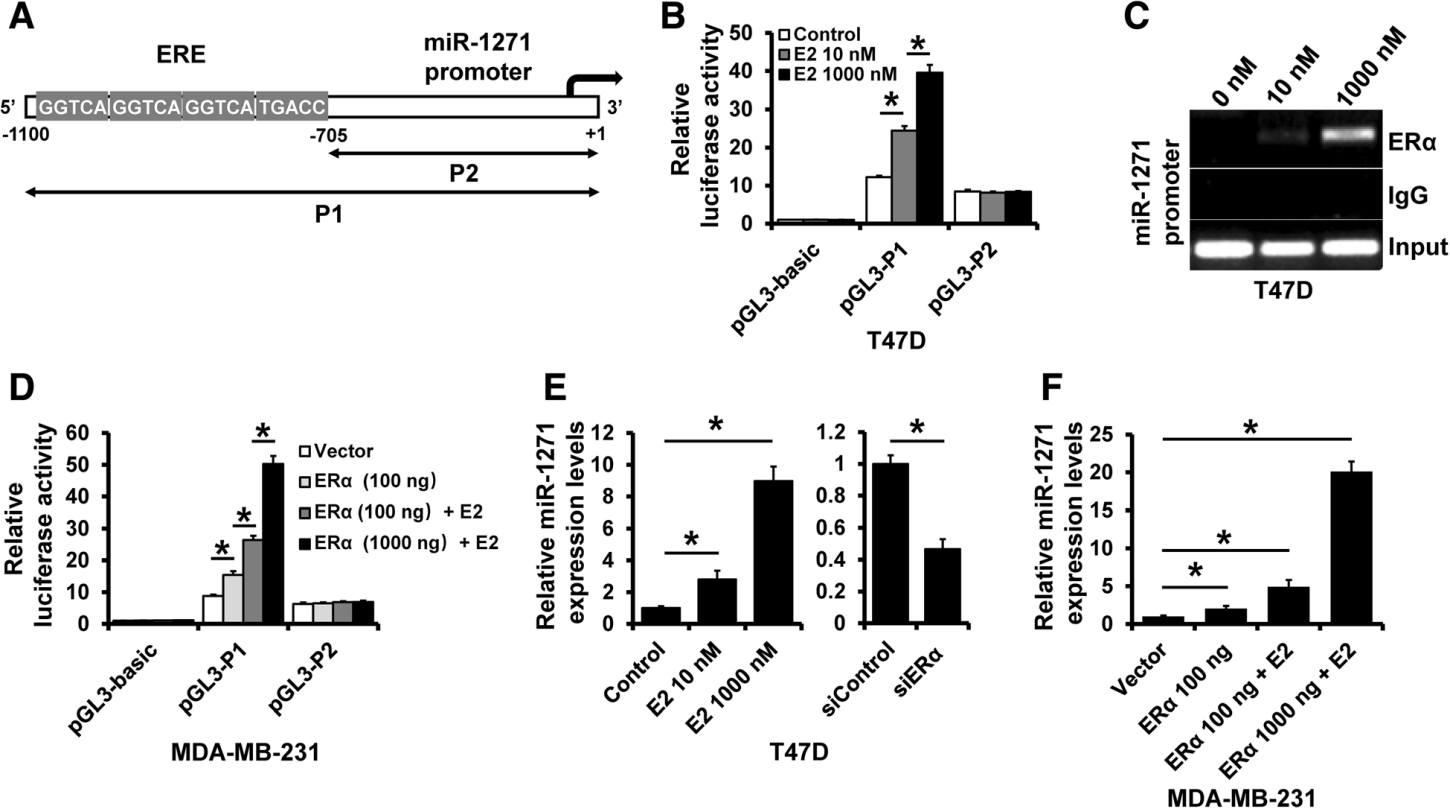
ERα transactivates the miR-1271 expression. **a** Promoter analysis of miR-1271. Four half EREs were located on the core promoter region of miR-1271 (− 1100 to + 1). **b** The miR-1271 promoter activity was measured in T47D cells with E2 treatment as determined by luciferase analysis. **c** Interaction between ERα and the miR-1271 promoter sequence in T47D cells with or without E2 treatment as determined by ChIP assay. **d** The miR-1271 promoter activity was measured in ERα-transfected MDA-MB-231 cells with or without E2 treatment as determined by luciferase analysis. **e** The expression of miR-1271 in T47D cells with E2 treatment (left), ERα-depleted T47D cells, as well as in control cells as determined by RT-qPCR. **f** The expression of miR-1271 in ERα-transfected MDA-MB-231 cells with or without E2 treatment as determined by RT-qPCR. *P < 0.05

We next examined the promoter sequence of miR-1271 and, interestingly, found four estrogen response elements (EREs) on the miR-1271 promoter region (Fig. 6a). Thus, we speculated that miR-1271 transcription is regulated by ERα. To confirm this, we constructed the miR-1271 promoter region with (P1) or without (P2) EREs into the pGL3-basic reporter, transfected it into ER+ T47D cells in addition to E2 stimulation. As shown in Fig. 6b, the T47D cells transfected with the pGL3-P1 showed high luciferase activity, which was further increased by addition of E2, while those transfected with pGL3-P2 showed low luciferase activity and E2 had no effect. ChIP assay revealed that ERα could bind to the miR-1271 promoter and binding was increased on addition of E2 in T47D cells (Fig. 6c). We next transfected pGL3-P1 or pGL3-P2 into ER- MDA-MB-231 cells in addition to ERα overexpression and E2. As shown in Fig. 6d, pGL3-P1 showed an increased luciferase activity after transfection with ERα plasmid, which was further increased by addition of E2, while pGL3-P2 showed no altered luciferase expression after transfection with ERα plasmid with or without E2 treatment. The expression of miR-1271 was also affected by E2 addition or transfection with siRNAs targeting ERα in T47D cells (Fig. 6e) or ERα plasmid in MDA-MB-231 cells (Fig. 6f). Together, these results indicated that ERα transactivates miR-1271 expression.

### SNAI2 suppresses miR-1271 expression by transcriptional down-regulation of ERα

**Fig. 7:**
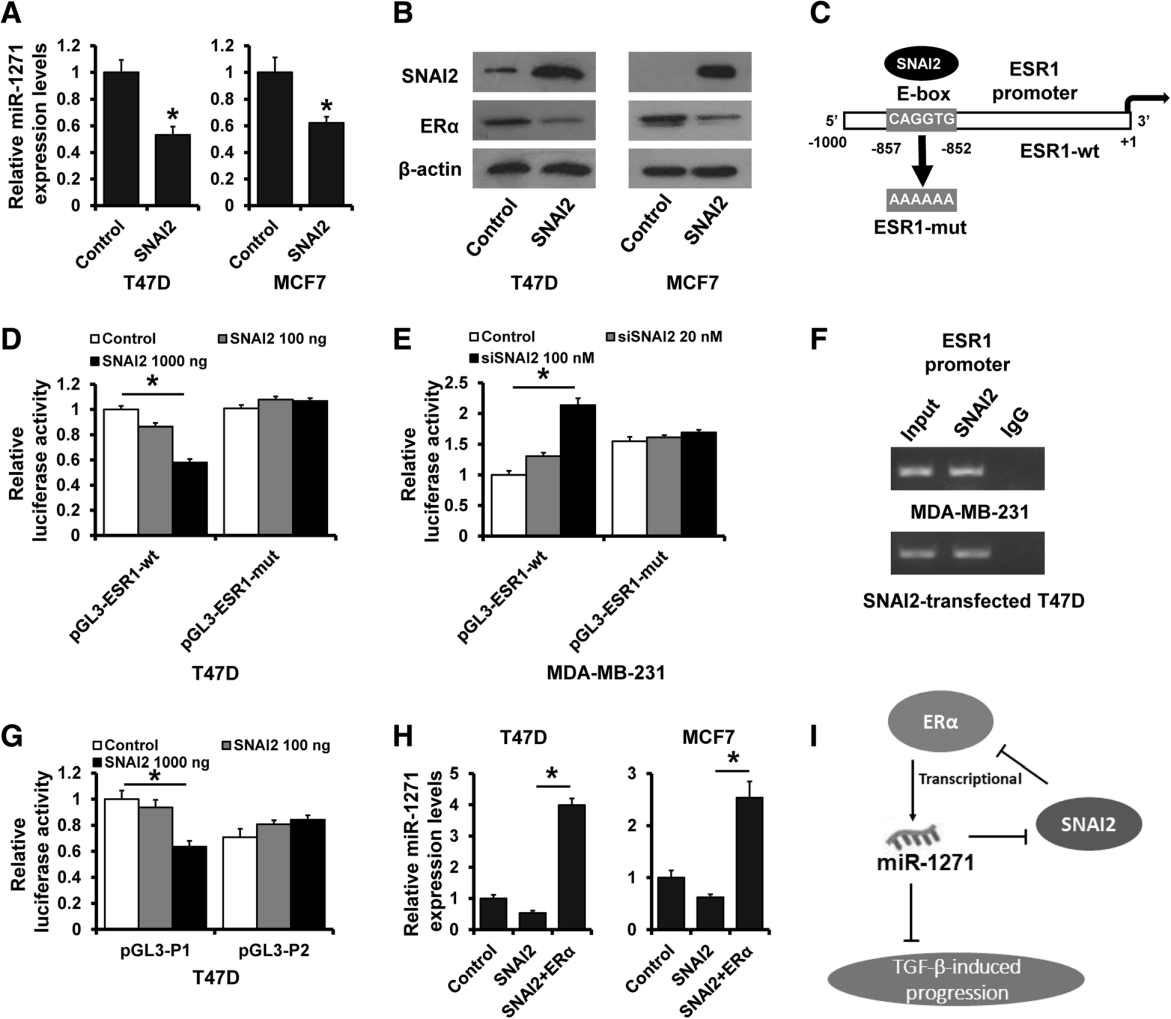
SNAI2 transcriptionally suppresses the ERα expression. **a** The expression of miR-1271 in SNAI2-transfected T47D cells (left) or MCF7 cells (right), as well as the control cells as determined by RT-qPCR. **b** The expression of SNAI2 and ERα in SNAI2-transfected T47D cells (left) or MCF7 cells, as well as the control cells as determined by western blotting. **c** Promoter analysis of ESR1. An E-box was located on the promoter region (− 1000 to + 1). **d** The ESR1 promoter activity was measured in SNAI2-transfected T47D and control cells as determined by luciferase analysis. **e** The ESR1 promoter activity was measured in MDA-MB-231 with transfection of siRNA targeting SNAI2 and control cells as determined by luciferase analysis. **f** Interaction between SNAI2 and the ESR1 promoter sequence in MDA-MB-231 cells (upper) or SNAI-transfected T47D cells (lower) as determined by ChIP assay. **g** The miR-1271 promoter activity was measured in SNAI2-transfected T47D and control cells as determined by luciferase analysis. **h** The expression of miR-1271 in SNAI2 or/and ERα-transfected T47D cells (left) or MCF7 cells (right), as well as the control cells as determined by RT-qPCR. **i** A model for the role of miR-1271 TGF-β-induced breast cancer progression

The expression of SNAI2 has been reported to be induced after TGF-β1 treatment in EMT [37]. Thus, we investigated whether SNAI2 could affect the miR-1271 expression in breast cancer cells. As shown in Fig. 7a, the miR-1271 expression was significantly decreased in SNAI2-overexpressed T47D or MCF7 cells compared with those of control cells by RT-qPCR. In addition, the expression of ERα was down-regulated in in SNAI2-overexpressed T47D or MCF7 cells compared with those of control cells by western blotting (Fig. 7b). We next examined the promoter sequence of ESR1 and, interestingly, found an E-box on the ERα promoter region (− 857 to − 852 bp; Fig. 7c). To further confirm the involvement of SNAI2 in ERα down-regulation, we conducted a dual-luciferase reporter assay in T47D or MDA-MB-231 cells. Transfection with different quality of SNAI2 plasmid (100 or 1000 ng) significantly decreased pGL3-ESR1-wt luciferase activity, but no significant change at pGL3-ESR1-mut was observed (Fig. 7d). In contrast, the luciferase activity was significantly increased in MDA-MB-231 cells transfection with different concentrations of siRNA targeting SNAI2 (20 or 100 nM). pGL3-ESR1-mut showed no altered luciferase expression after transfection with siRNAs targeting SNAI2 (Fig. 7e). To address whether SNAI2 can bind to E-box in the ESR1 promoter, ChIP assay was carried out in MDA-MB-231 cells expressing high level of endogenous SNAI2 and SNAI2-transfected T47D cells. The results showed that SNAI2 bound to E-box on the ESR1 promoter region (Fig. 7f). To further demonstrate that SNAI2 down-regulates miR-1271 expression by transcriptionally suppressing ESR1, we conducted a dual-luciferase reporter assay in T47D cells by using pGL3-P1 and pGL3-P2. Transfection with different quality of SNAI2 plasmid (100 or 1000 ng) significantly decreased pGL3-P1 luciferase activity, but no significant change at pGL3-P2 was observed (Fig. 7g). Furthermore, overexpression of ERα eliminates the effect of SNAI2 overexpression on the miR-1271 expression (Fig. 7h). Collectively, our data support the conclusion that SNAI2 suppresses miR-1271 expression by transcriptional down-regulation of ERα.

## Discussion

In the present study, we identified that miR-1271 as a tumor suppressor and an EMT inhibitor both in vitro and in vivo, and correlates with survival in patients with Luminal A breast cancer. Moreover, SNAI2 was found to be a direct target of miR-1271 and a transcriptional repressor on regulation of ERα expression. Furthermore, depletion of miR-1271 reverses the suppressive effect of estrogen or ERα on TGF-β-induced EMT. Therefore, our results revealed that ERα-miR-1271-SNAI2 feedback loop plays an important role in breast cancer progression through regulating TGF-β-induced EMT and demonstrated that miR-1271 functions as a tumor-suppressive miRNA in breast cancer.

miRNAs are small, non-protein coding RNAs first identified over a decade ago, and their dysregulation has been implicated in cancer development and progression. Although much more studies have indicated that miR-1271 functions as a tumor suppressor in many types of human cancers [13–33], the mechanism of miR-1271 in breast cancer progression is still limited. In this study, we demonstrated that miR-1271 suppresses breast cancer progression both in vitro and in vivo. Metastasis, a major obstacle to successful cancer therapy, is a multi-step process where cancer cells leave the primary tumor, enter the vasculature, survive in the circulation, disseminate to distant sites, and form secondary tumors [38]. EMT has been shown to play pivotal roles in these steps to promote metastasis. During EMT, epithelial cells lose their junctions and apical–basal polarity, reorganize their cytoskeleton, and acquire an invasive mesenchymal phenotype. The gain of mesenchymal markers, vimentin and N-cadherin and loss of epithelial marker E-cadherin, are often observed when EMT occurs [39]. Similar to the observation in hepatocellular carcinoma [13], pancreatic cancer [21] and gastric cancer [17], we found that miR-1271 reduced the expression of vimentin and N-cadherin and induced the expression of E-cadherin in breast cancer cells, whereas depletion of endogenous miR-1271 by its inhibitors yielded the opposite effects, suggesting that miR-1271 is an EMT suppressor in breast cancer.

TGF-β, a key driver of EMT, induces the expression of vimentin and N-cadherin and represses E-cadherin expression, thus promoting EMT and cancer metastasis [40]. ERα has been identified to promote the growth of primary breast cancer, however, it can also antagonize TGF-β signaling pathway that lead to EMT [4, 5]. ERα- tumors are more likely to be of higher histological grade than more differentiated ERα + tumors. Patients with these tumors had an unfavorable outcome compared to the ERα + breast cancer patients. These results raise the possibility that ERα has biphasic effects on breast cancer progression. Here, we demonstrated that ERα transactivates the miR-1271 expression in an estrogen-dependent manner. Furthermore, miR-1271 reverses the suppressive effect of estrogen on TGF-β-induced EMT. These results indicated that miR-1271 down-regulation is required for TGF-β-induced EMT in ERα + breast cancer cells. In this study, we found that miR-1271 as a predictor in patients with Luminal A breast cancer. This may cause by a lower or loss of miR-1271 expression in other subtypes of breast cancer.

SNAI2 (also known as Slug), a member of the SNAIL family, is a C2H2-type zinc finger transcription factor involved in the regulation of cell differentiation fate and determination [41]. SNAI2 plays an essential role in development and cancer-associated EMT. SNAI2 has a highly conserved zinc finger structures, allowing DNA binding at the E-box in the promoter region of target genes, such as E-cadherin [42]. Aberrant expression of SNAI2 has been observed in many human cancers, including breast cancer [43]. ERα appears to downregulate SNAI2 expression by repression of SNAI2 transcription or induction of SNAI2 degradation [3]. Here, we found that SNAI2 functions as a transcriptional repressor, which depends on the E-box binding sites on the ESR1 promoter region. Our results showed that SNAI2 binds to the ESR1 promoter and transcriptionally suppresses ERα expression. Interestingly, we also demonstrated that SNAI2 is a direct target of miR-1271 and miR-1271 suppresses breast cancer progression and EMT phenotype by targeting SNAI2. Therefore, our study reveals an ERα-miR-1271-SNAI2 feedback loop in regulation of TGF-β signaling during breast cancer development and progression.

## Conclusions

In summary, we demonstrated that miR-1271 is a tumor suppressor in breast cancer and also a predictor in patients with Lumina A breast cancer. miR-1271 suppresses breast cancer progression and EMT phenotype by targeting SNAI2. Furthermore, miR-1271 is a transcriptional target of ERα and ESR1 is a transcriptional target of SNAI2. Based on the findings from this study and others, we propose a model that highlights the role of miR-1271 in regulating TGF-β signaling during ERα + breast cancer progression (Fig. 7i). The uncovering of this ERα-miR-1271-SNAI2 feedback loop will extend our comprehension of TGF-β network complexity and miR-1271 may serve as a new option to target TGF-β signaling for breast cancer intervention.

## Additional file

Additional file 1: **Figure S1.** miR-1271 does not affect cell proliferation in breast cancer. A and B, MTT (A) and colony formation (B) analysis of cell proliferation in T47D or MCF7 cells transfected with miR-1271, as well as in control cells. C and D, MTT (C) and colony formation (D) analysis of cell proliferation in miR-1271-expressing MDA-MB-231 cells, as well as in control cells. (DOCX 166 kb)

## Abbreviations

ChIP: chromatin immunoprecipitation
EMT: epithelial–mesenchymal transition
ERE: estrogen response element
ERα: estrogen receptor-α
ESR1: Estrogen Receptor 1
PBS: phosphate buffered saline
RT-qPCR: reverse transcription quantitative polymerase chain reaction
siRNA: small interfering RNA
SNAI2: Snail family transcriptional repressor 2
TGF-β: transforming growth factor-β
UTR: untranslated region
