# Supplementary material for: Estrogen receptor-α-miR-1271-SNAI2 feedback loop regulates transforming growth factor-β-induced breast cancer progression
Source: J Exp Clin Cancer Res. 2019 Mar 1;38:109. doi: 10.1186/s13046-019-1112-4 (PMC6397493; doi:10.1186/s13046-019-1112-4)
Supplement: Supplementary file 1 — Figure S1. miR-1271 does not affect cell proliferation in breast cancer. A and B, MTT (A) and colony formation (B) analysis of cell proliferation in T47D or MCF7 cells transfected with miR-1271, as well as in control cells. C and D, MTT (C) and colony formation (D) analysis of cell proliferation in miR-1271-expressing MDA-MB-231 cells, as well as in control cells. (DOCX 166 kb) [file 13046_2019_1112_MOESM1_ESM.docx]

**Supplemental data**

**
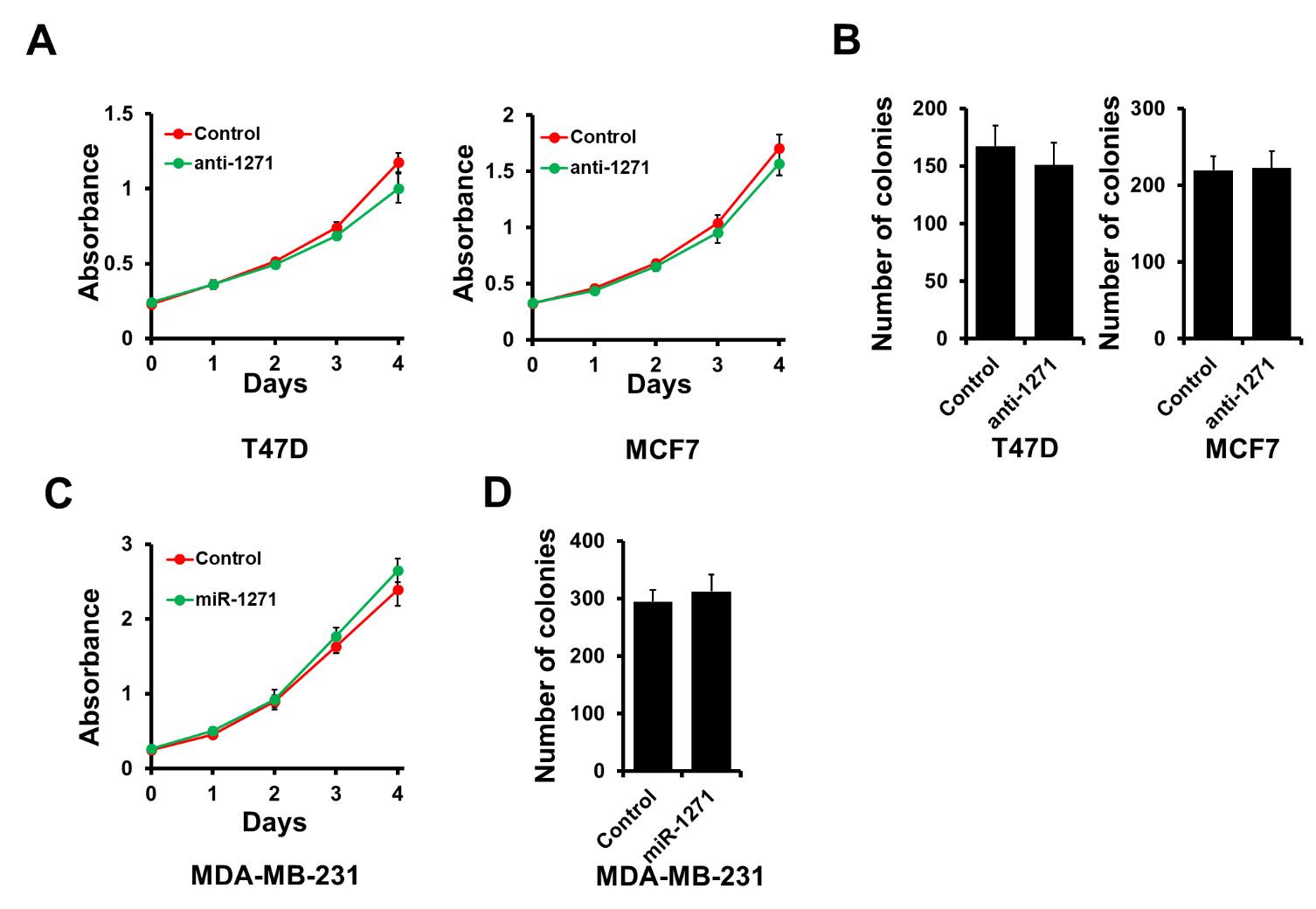
**

**Figure S1. miR-1271 does not affect cell proliferation in breast cancer. A and B,** MTT (A) and colony formation (B) analysis of cell proliferation in T47D or MCF7 cells transfected with miR-1271, as well as in control cells. **C and D,** MTT (C) and colony formation (D) analysis of cell proliferation in miR-1271-expressing MDA-MB-231 cells, as well as in control cells.
